# The median Effective Dose (ED_50_) and the 95% Effective Dose (ED_95_) of alfentanil in inhibiting responses to cervical dilation when combined with ciprofol during hysteroscopic procedure: a prospective, double-blind, dose-finding clinical study

**DOI:** 10.1186/s12871-025-03217-5

**Published:** 2025-07-19

**Authors:** Run Gao, Shu-Xi Li, Yan-Hong Zhou, Li Xing, Jin-Peng Fu, Jian-Jun Shen, Xin-Zhong Chen, Li-Li Xu

**Affiliations:** 1 Department of Anesthesia, Suzhou Municipal Hospital, Suzhou, Jiangsu Province China; 2 Department of Anesthesia, Women’s Hospital, Zhejiang University School of Medicine, No.1 Xueshi Road, Hangzhou, Zhejiang Province 310006 China; 3 Department of Anesthesia, Hangzhou Linping Maternal and Child Health Hospital, Hangzhou, Zhejiang Province China; 4 Department of Anesthesia, Jinhua municipal central hospital, Jinhua, Zhejiang Province China; 5 Department of Anesthesia, the Second Affiliated Hospital, Zhejiang University School of Medicine, Hangzhou, Zhejiang Province China

**Keywords:** Alfentanil, Ciprofol, Cervical dilation, Hysteroscopy

## Abstract

**Background:**

Alfentanil, a short-acting µ opioid receptor agonist, has recently been confirmed that when combined with propofol for daytime hysteroscopy, it provided more stable hemodynamics compared with sufentanil, and has a lower incidence of hypoxemia and postoperative nausea and vomiting. The object of the trial was to determine the median effective dose (ED_50_) and the 95% effective dose (ED_95_) of alfentanil in inhibiting responses to cervical dilation when combined with ciprofol and explore the effect of alfentanil on reducing ciprofol requirement during hysteroscopy.

**Methods:**

One hundred and forty patients scheduled hysteroscopy under monitored anesthesia care were randomized to receive a bolus of 8 µg·kg^−1^, 10 µg·kg^−1^, 12 µg·kg^−1^, 14 µg·kg^−1^ intravenous alfentanil or 0.15 µg⋅kg^−1^ intravenous sufentanil followed by a bolus of 0.5 mg·kg^−1^ ciprofol. Whether there was loss of response to cervical dilation or not in each patient was recorded. We used the probit analysis to determine ED_50_ and ED_95_ of alfentanil in inhibiting responses to cervical dilation when combined with ciprofol. The requirement of ciprofol, the emergence time, the visual analogue scale score of pain at five time points, and the incidence of adverse events were recorded.

**Results:**

The calculated ED_50_ and ED_95_ of alfentanil were 9.73 [95% CI 8.57 to 10.56] µg·kg^−1^ and 15.02 [95% CI 13.57 to 18.12] µg·kg^−1^, respectively. Ciprofol requirements were lower in patients given 10 µg·kg^−1^ (0.795 [ 0.707 to 0.889] mg·kg^−1^), 12 µg·kg^−1^ (0.799 [0.601 to 0.913] mg·kg^−1^), and 14 µg·kg^−1^ (0.789 [0.660 to 0.968] mg·kg^−1^) alfentanil than those given 8 µg·kg^−1^ alfentanil (1.082 [ 0.853 to 1.271] mg·kg^−1^) alfentanil and 0.15 µg⋅kg^−1^ sufentanil (1.046 [0.861 to 1.427] mg·kg^−1^) (*P* < 0.001). Emergence time was lower in patients given 10 µg·kg^−1^ (0.9 [0.8 to 1.2] min), 12 µg·kg^−1^ (0.8 [0.6 to 1.0] min) than those given 8 µg·kg^−1^ (1.9 [1.0 to 2.8] min) and 14 µg·kg^−1^ (1.5 [1.0 to 2.3] min) alfentanil, and 0.15 µg·kg^−1^ sufentanil (1.4 [1.0 to 2.0] min) (*P* < 0.001). The visual analogue scale score of pain at the time of 30 min and 1 h after surgery was lower in patients given 10 µg·kg^−1^, 12 µg·kg^−1^, and 14 µg·kg^−1^ alfentanil when compared with 8 µg·kg^−1^ alfentanil and 0.15 µg⋅kg^−1^ sufentanil (*P* < 0.001). The incidence of intraoperative hypotension was lower in patients given 8 µg·kg^−1^, 10 µg·kg^−1^, 12 µg·kg^−1^ alfentanil, and 14 µg·kg^−1^ alfentanil than those given 0.15 µg·kg^−1^ sufentanil (*P =* 0.044), while the incidence of intraoperative desaturation was higher in patients given 14 µg·kg^−1^ alfentanil than those given 8 µg·kg^−1^, 10 µg·kg^−1^, and 12 µg·kg^−1^ alfentanil, and 0.15 µg·kg^−1^ sufentanil (*P* < 0.001).

**Conclusions:**

For women undergoing hysteroscopic surgery, a dose of 10–12 µg·kg^−1^ of alfentanil was associated with significant ciprofol-sparing effect, earlier anesthesia emergence, better postoperative analgesia, and less unexpected hemodynamic events compared with sufentanil, but 14 µg·kg^−1^ alfentanil had the risk of transient desaturation and delayed anesthesia recovery. Indications and the optimal dose of alfentanil in this patient population need further clarification.

**Trial registration:**

The study was then registered on January 24, 2024 at the Chinese Clinical Trial Registry which participates in the World Health Organization International Clinical Trials Registry Platform (Identifier: ChiCTR2400080232) before enrolling the first participant and written informed consent was obtained by each patient.

**Supplementary Information:**

The online version contains supplementary material available at 10.1186/s12871-025-03217-5.

## Introduction

As is well known, some common gynecological diseases such as intrauterine adhesions, endometrial polyps, abnormal uterine bleeding, uterine malformations and other intrauterine pathological conditions have disturbed normal fertility and even impair physical and mental health in women of childbearing age [1, 2]. Compared with medication and other treatments, hysteroscopy has become one of the ideal methods for diagnosis and treatment of these pathological state. Meanwhile, the monitored anesthesia care (MAC) using opioid drugs combined with hypnotic and sedative drugs has become the main anesthesia method that is superior to epidural anesthesia or spinal anesthesia [3, 4]. However, almost all of the current conventional µ opioid receptor agonists, such as fentanyl or sufentanil can increase the rate of emergence-treatment adverse effects such as itching, excitement, dizziness, blurred vision, nausea, vomiting, biliary sphincter spasms, sweating, constipation, urinary retention, and delayed recovery [5]. Unfortunately, some serious adverse events can occur especially when rapidly intravenous administrated, such as respiratory depression, asphyxia, laryngeal spasms, hypotension, bradycardia, chest wall muscle stiffness, etc. Hence, the search for an alternative drug to reduce the above mentioned side-effects becomes prudent during hysteroscopic surgery.

Alfentanil is a novel potent opioid agent with 15 times analgesic intensity of morphine and has more rapid analgesic onset (30s) and time to peak effect (1.4 min), shorter distribution and elimination half-lives (15 min) as well as smaller volume of distribution and total body clearance compared with fentanyl and sufentanil [6, 7]. Therefore, it is suitable for the induction and maintenance of anesthesia during relatively short surgeries and outpatient treatments [8]. A previous clinical trial declared that in patients undergoing coronary artery bypass graft surgery (CABG), those anaesthetized with alfentanil or sufentanil exhibited a shorter time for the plasma concentration to decrease by 80% (t80) after cessation of the infusion, and needed less time of mechanical ventilatory support, with more predictable time to tracheal extubation than those receiving fentanyl combined with propofol [9]. In another prospective, randomized, double-blind study of healthy surgical patients aged 16 to 65 years, alfentanil 10 µg·kg^−1^ similarly effectively attenuated the pressor response to nasotracheal intubation, compared with remifentanil 1 µg·kg^−1^ given over 30 s, followed by a remifentanil infusion of 0.5 µg·kg^−1^·min^−1^, but the incidence of hypotension in patients administered alfentanil was lower [10]. Interestingly, a recent randomized controlled trial furtherly reported that single-use of 10 µg·kg^−1^ alfentanil significantly lowered the incidence of hypotension and cognitive dysfunction, and shortened discharge time when compared with 2 mg·kg^−1^ propofol for patients undergoing elective colonoscopy [11]. Importantly, a recent randomized clinical trial of daytime hysteroscopy in Chinese patients demonstrated that when combined with target-controlled infusion (TCI) of propofol, alfentanil at a dose of 10 µg·kg^−1^ was safer than sufentanil at a dose of 0.15 µg·kg^−1 ^[12]. However, scanty availble literatures paid more attention on median effective dose (ED_50_) and the 95% effective dose (ED_95)_ of alfentanil in inhibiting responses to cervical dilation when combined with ciprofol and the effect of alfentanil on the ciprofol-sparing during hysteroscopy. Subsequently, we carried out the prospective, double-blinded, randomized study to explore the ED_50_ and ED_95_ of alfentanil in inhibiting responses to cervical dilation when combined with ciprofol and investigate the effect of alfentanil on the requirement of ciprofol requirement during hysteroscopy.

## Methods

### Study design and subjects

Our research was approved by the Research Ethics Committee of Women’s Hospital, Zhejiang University School of Medicine (IRB-20230325-R; September 28, 2023). The protocol was registered at the Chinese Clinical Trial Registry (https://www.chictr.org.cn; Identifier: ChiCTR2400080232; Principal investigator: Li-Li Xu; January 24, 2024). The study was then conducted at the Women’s Hospital Affiliated to Zhejiang University College of Medicine in Hangzhou, Zhejiang Province, China, from January 31th, 2024, to March 1th, 2024, with written informed consent from all included patients. The work was reported in line with Consolidated Standards of Reporting Trials (CONSORT) Guidelines [13]. 

One hundred and forty female patients, American Society of Anesthesiologists (ASA) grade I or II, aged 18–65 years who scheduled for hysteroscopic surgery, were included in our study. Patients with body mass index (BMI) > 24 kg·m^−2^, pregnancy, a history of cardiopulmonary disease, severe neuropsychiatric conditions, renal or liver dysfunction, obstructive sleep apnea, or a history of opioid use, inhalation risk factors, opioid allergy, or contraindications were excluded from our study.

### Randomization and blinding

Patients were randomly assigned to receive either intravenous alfentanil of 8 µg·kg^−1^ (Group A-8), 10 µg·kg^−1^ (Group A-10), 12 µg·kg^−1^ (Group A-12), 14 µg·kg^−1^ (Group A-14) [12, 14, 15] or 0.15 µg⋅kg^−1^ (Group S) intravenous sufentanil [16] according to a computer-generated random order reserving in a sealed, opaque envelope. Afterwards, the researcher (LX) opened the envelope, prepared the research drug with the same 10 ml syringe and marked the research serial number. The researchers who collected and kept the data, the attending anesthesiologists (ZY and LS), and the patients were all blinded to our preparation.

### Anesthesia management and intervention

Routine fasting was administered to each patient before the operation, but no preoperative medication was given. After the patients entered the operating room and a peripheral venous access was established, a multi-parameter monitor was used to monitor electrocardiogram (ECG), pulse oximeter (SpO_2_), noninvasive blood pressure (NIBP), end-expiratory partial pressure of carbon dioxide (P_ET_CO_2_), and state entropy (SE). At the same time, the patients were provided with supplemental oxygen 5 L·min^−1^.

While the surgical site was being especially cleaned, the corresponding dose of alfentanil or sufentanil was intravenously administrated according to the group allocation, followed by an initial dose of 0.5 mg·kg^−1^ ciprofol. After the patients lost consciousness (LOC, loss of response to speech commands and eyelash reflexes), and SE was between 40 and 60, the gynecologist began to dilate the cervix with a probe. If the patients did not show body movement, coughing or eye opening, a negative cervical dilation response was judged. On the contrary, if the patients showed body movement, coughing, or eye opening, a positive cervical dilation response was judged, and then a bolus of 0.2 mg·kg^−1^ ciprofol was given every 4 min. Furthermore, if the cervical dilation response still could not be completely inhibited after administration of 0.2 mg·kg^−1^ ciprofol three times, 0.1 µg⋅kg^−1^ sufentanil was given every 10 min. Whether there was loss of response to cervical dilation of each patient were all recorded. A Probit regression model was used to calculate the ED_50_ and ED_95_ of alfentanil in inhibiting responses to cervical dilation when combined with ciprofol according to the effective number of patients. During the operation, continuous intravenous infusion of 0.5 mg·kg^−1^·h^−1^ ciprofol was administered to keep the entropy index between 40 and 60, and the patients were sent to the post-anesthesia care unit (PACU) after recovery.

### Data collection and outcome assessment

Intraoperative data included the total dose of ciprofol and the emergence time from anesthesia (defined as the duration between discontinuation of ciprofol and spontaneous opening of eyes) [16]. The Steward score [17] (including the degree of alertness, the degree of airway patency, and the degree of limb range of motion) was used to assess the conscious or unconscious state of the patients during the period from ciprofol discontinuation to spontaneous eye opening. A total score greater than 4 points was determined as conscious. We assessed the pain score using the visual analogue scale (VAS, 0–10 points representing different degrees of pain, 0 = no pain, 10 = severe pain) at the time of 30 min, 1 h, 6 h, 12 h, and 1 d after surgery. While sedation level was assessed by Ramsay sedation scale (1 = restlessness; 2 = completely awake, quiet and cooperative; 3 = drowsiness but responding to verbal commands; 4 = light asleep but responding to touch or pain; 5 = asleep but slowly responding to touch or pain; 6 = deeply asleep and does not respond) at the time of 30 min, 1 h and 6 h after surgery.

Systolic blood pressure (SBP), diastolic blood pressure (DBP), heart rate (HR), and SpO_2_ were measured every 5 min. Postoperative data included use of NSAIDs, opioids, and other painkillers, overall satisfaction score, Modified Aldrete score, length of PACU stay and hospital stay after surgery, postoperative complications [18]. All the intraoperative and postoperative side effects including bradycardia, tachycardia, hypotension, hypertension, desaturation, nausea and vomiting, shivering, myotonia, abdominal bloating, constipation, and pruritus were observed and managed by routine practice. Bradycardia was defined as a HR less than 50 beats⋅min^−1^, and tachycardia was defined as a HR more than 100 beats⋅min^−1^. Hypotension is defined as SBP less than 90 mmHg or 80% of baseline, and hypertension is defined as SBP more than 140 mmHg or 20% of baseline. Desaturation is defined as a condition where SpO_2_ is less than 95%.

The primary outcome was ED_50_ and ED_95_ of alfentanil in inhibiting responses to cervical dilation when combined with ciprofol, which were calculated based on the effective number of patients of inhibiting the responses to cervical dilation (including body movement, coughing, and eye opening) after the administration of alfentanil combined with ciprofol by using Probit regression. The secondary outcomes included the ciprofol requirement, the emergence time, the VAS score of pain on postoperative 30 min, 1 h, 6 h, 12 h and day 1. The other outcomes included Ramsay sedation scale on postoperative 30 min, 1 h, 6 h, 12 h and day 1.

### Statistical analysis

Sample size was estimated using PASS (version 11.0.7; NCSSLLC, Kaysville, UT), the calculation was based on the results of our pre-experiment that the success ratios of ciprofol combined sufentanil group (control group) and ciprofol combined afentanil group at the dose of 8 µg⋅kg^−1^, 10 µg⋅kg^−1^, 12 µg⋅kg^−1^, and 14 µg⋅kg^−1^ were 30%, 40%, 40%, 60%, and 80%, respectively. Considering 90% power, a significance level of 0.05, and possible dropouts, a sample size of 140 patients (28 patients per group) would be sufficient.

The normal ditribution of continuous variables were examined using the Kolmogorov-Smirnov test. Normality distributed continuous variables were presented as mean ± standard deviation (SD), and compared using one-way analysis of variance (ANOVA) between groups, while skewed distributed continuous variables were presented as the median and inter-quartile range (IQR) and compared using the Kruskal-Wallis test between groups. Categorical variables were presented as numbers (%), and analyzed using the Mantel-Haenszel Chi-square test (the test for linear trend). If an overall test of difference among groups was significant, Chi-square test was used for pairwise comparisons. For each group, we performed probit regression analysis through grouping methods calculating the tallied numbers of ineffectiveness and effectiveness of each dose and obtained estimates of the ED_50_ and ED_95_ for alfentanil and computed the relative mean potency with 95% CI [19, 20]. Kaplan-Meier log-rank survival analysis was performed to compare the cumulative probability of the emergence time after discontinuation, and calculated the ciprofol requirement (mg·kg^−1^·min^−1^) divided by MAC duration (from induction to discontinuation of ciprofol) and body weight.

Statistical analysis and drawing graphics were performed with IBM SPSS for Windows version 22.0 (IBM Corp, Armonk, NY, USA) and GraphPad Prism version 5.0 (GraphPad Software Inc., San Diego, CA, USA). Two side *P* values < 0.05 were set as be statistically significant.

## Results

### Patient characteristics

**Fig. 1:**
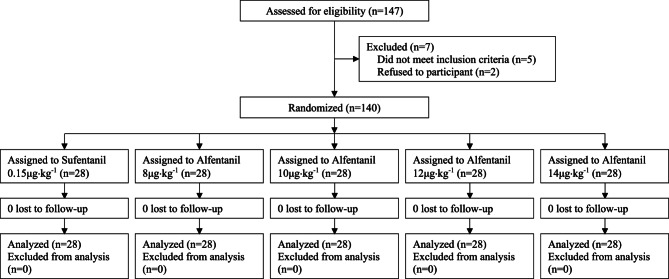
Consolidated Standards of Reporting Trials (CONSORT) flow diagram defining patient assessment and enrollment numbers in the study

One hundred and forty-seven patients were screened for eligibility, five patients did not meet the inclusion criteria and two patients declined to participate. A total of 140 patients were randomized into five groups (*n* = 28 each) and included in the final analysis as shown in Consort Flow Diagram (Fig. 1). Baseline demographic and surgical characteristics were comparable between groups as shown in Table 1.

**Table 1:** Patient characteristics

	Sufentanil 0.15 μg×kg^-1^(*n*=28)	Alfentanil 8 μg×kg^-1^ (*n*=28)	Alfentanil 10 μg×kg^-1^ (*n*=28)	Alfentanil 12 μg×kg^-1^ (*n*=28)	Alfentanil 14 μg×kg^-1^ (*n*=28)	***P*-value**
Age (yr)	33.0 (28.3, 38.8)	32.5 (27.5, 36.5)	33.0 (30.3, 37.0)	31.5 (29.0, 32.8)	32.0 (31.0, 35.0)	0.599
Height (cm)	160.1 (158.0, 164.0)	159.5 (155.3, 162.0)	160.5 (158.0, 165.8)	160.5 (159.3, 163.0)	159.0(156.0, 163.0)	0.145
Weight (kg)	56.5 (47.3, 61.4)	55.0 (51.1, 60.0)	57.0 (54.3, 62.9)	55.0 (50.5, 59.0)	53.5 (48.9, 59.0)	0.340
BMI (kg·m^-2^)	21.7 (18.9, 23.5)	22.5 (20.5, 23.7)	22.2 (20.3, 23.5)	20.8 (19.8, 23.4)	20.7 (19.7, 23.3)	0.259
Type of hysteroscopic surgery						0.637
Polypectomy	16 (57)	15 (54)	11 (39)	14 (50)	12 (41)	
Lysis of adhesion	12 (43)	13 (46)	15 (61)	12 (50)	15 (59)	
Surgery duration (min)	20.0 (14.3, 27.3)	18.5 (13.0, 24.5)	20.0 (16.0, 25.0)	17.5 (12.3, 22.3)	19.5 (12.3, 30.0)	0.518

Data are presented as median (inter-quartile range) or N (%)

*BMI* Body mass index

### Efficacy outcomes

**Fig. 2:**
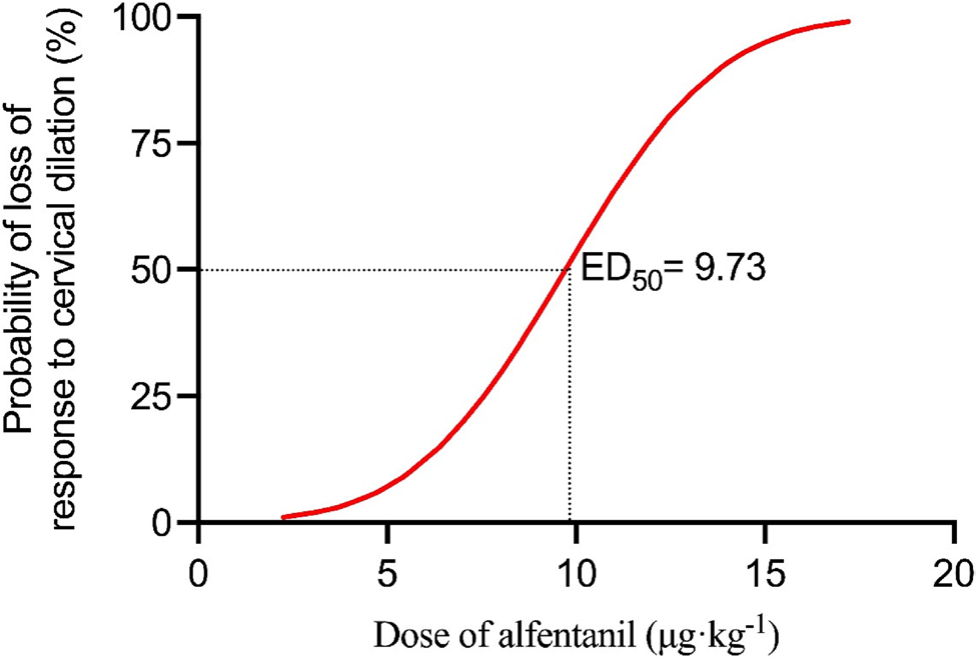
Dose-response curve of ciprofol infusions for loss of response to cervical dilation plotted estimated probabilities of success (1%-100%) versus the dose of alfentanil (μg·kg^-1^) using probit regression analysis

**Fig. 3:**
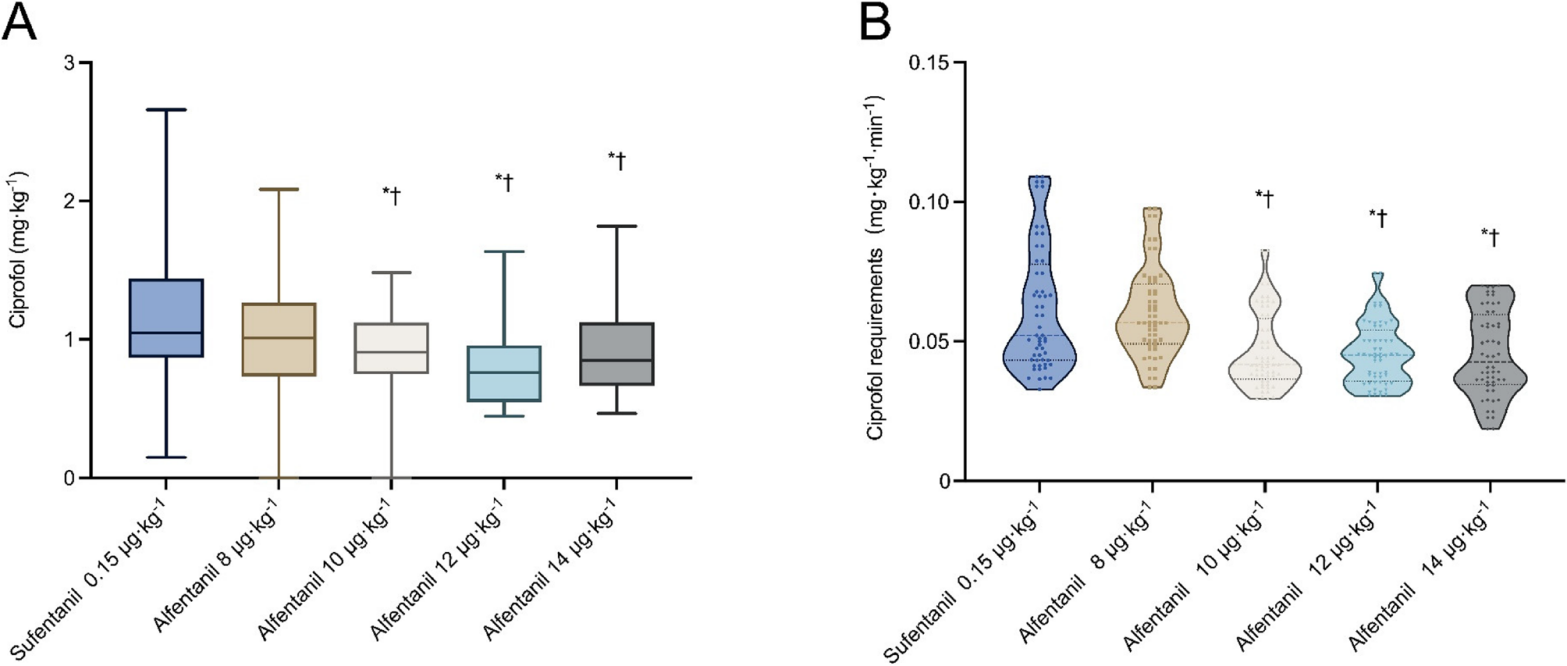
**A** Ciprofol requirement (mg·kg^-1^) during the procedure at doses of 8 μg·kg^-1^, 10 μg·kg^-1^, 12 μg·kg^-1^, 14 μg·kg^-1^ alfentanil and 0.15mg×kg^-1^ sufentanil. *P<0.005, compared with sufentanil 0.15mg×kg^-1^ after Bonferroni correction. †*P*<0.005, compared with alfentanil8 μg·kg^-1^ after Bonferroni correction. **B** Ciprofol requirement (mg·kg^-1^·min^-1^) during the procedure at doses of 8 μg·kg^-1^, 10 μg·kg^-1^, 12 μg·kg^-1^, 14 μg·kg^-1^ alfentanil and 0.15mg×kg^-1^ sufentanil. *P<0.005, compared with sufentanil 0.15mg×kg^-1^ after Bonferroni correction. †*P*<0.005, compared with alfentanil8 μg·kg^-1^ after Bonferroni correction

**Fig. 4:**
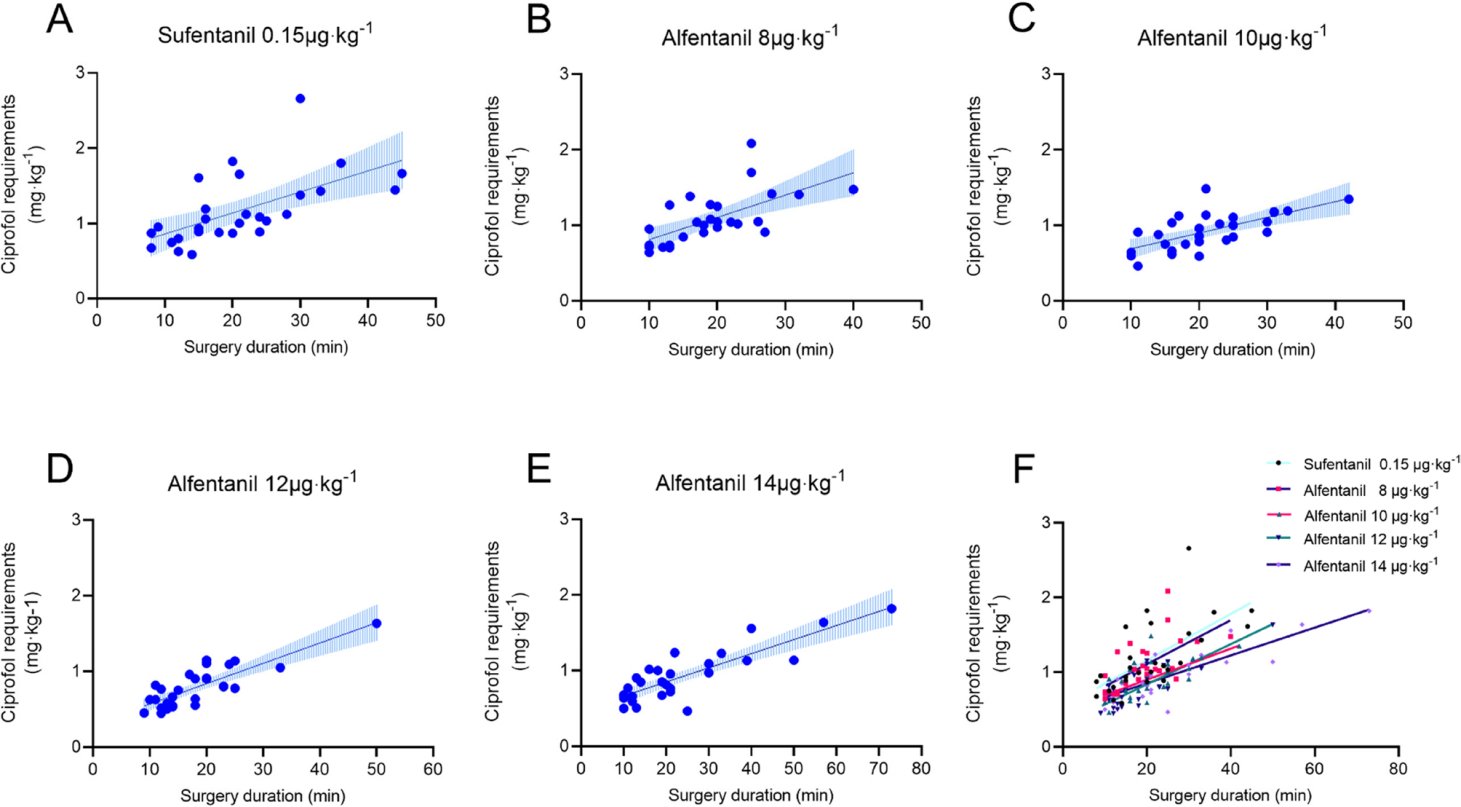
**A**-**F** The slopes of the ciprofol requirements-duration curves were 0.029 (95% CI 0.016 to 0.043), 0.021 (95% CI 0.011 to 0.030), 0.026 (95% CI 0.019 to 0.034), 0.019 (95% CI 0.014 to 0.023), 0.031 (95% CI 0.017 to 0.046) at doses of 8 μg·kg^-1^, 10 μg·kg^-1^, 12 μg·kg^-1^, 14 μg·kg^-1^ alfentanil and 0.15mg×kg^-1^ sufentanil, respectively

The calculated ED_50_ and ED_95_ of alfentanil were 9.73 [95% CI 8.57 to 10.56] µg·kg^−1^ and 15.02 [95% CI 13.57 to 18.12] µg·kg^−1^, respectively (Table 2). Effective number of patients was higher in patients given 10 µg·kg^−1^, 12 µg·kg^−1^, and 14 µg·kg^−1^ alfentanil than those given 8 µg·kg^−1^ alfentanil (*P* < 0.001; Table 2). Dose response curves derived from probit regression analysis are shown in Fig. 2. Ciprofol requirements was lower in Group A-10 (0.795 [0.707 to 0.889] mg·kg^−1^), Group A-12 (0.799 [0.601 to 0.913] mg·kg^−1^), and Group A-14 (0.789 [0.660 to 0.968] mg·kg^−1^) than those in Group A-8 (1.082 [0.853 to 1.271] mg·kg^−1^) and Group S (1.046 [0.861 to 1.427] mg·kg^−1^) (*P* < 0.001; Table 2; Fig. 3). Similarly, the ciprofol requirements was lower in Group A-10 (0.042 [0.031 to 0.049] mg·kg^−1^·min^−1^), Group A-12 (0.045 [0.038 to 0.050] mg·kg^−1^·min^−1^), and Group A-14 (0.042 [0.033 to 0.056] mg·kg^−1^·min^−1^) than those in Group A-8 (0.058 [0.052 to 0.068] mg·kg^−1^·min^−1^) and Group S (0.056 [0.049 to 0.073] mg·kg^−1^·min^−1^) (*P* < 0.001; Table 2). The slope of the ciprofol requirements-duration curves was lower in Group A-10 (0.021 [95% CI: 0.011 to 0.030]), Group A-12 (0.026 [95% CI: 0.019 to 0.034]), and Group A-14 (0.019 [95% CI: 0.014 to 0.023]) than those in Group A-8 (0.029 [95% CI: 0.016 to 0.043]) and Group S (0.031 [95% CI: 0.017 to 0.046]) (*P* < 0.001; Table 2; Fig. 4).

**Table 2:** Efficacy outcomes

	Sufentanil 0.15 μg×kg^-1^ (*n*=28)	Alfentanil 8 μg×kg^-1^ (*n*=28)	Alfentanil 10 μg×kg^-1^ (*n*=28)	Alfentanil 12 μg×kg^-1^ (*n*=28)	Alfentanil 14 μg×kg^-1^ (*n*=28)	***P*-value**
Primary outcome						
ED_50_		9.73 (8.57 to 10.56)				
ED_95_		15.02 (13.57 to 18.12)				
Secondary outcomes						
Time to anesthesia emergence (min)	1.4 (1.0, 2.0)	1.9 (1.0, 2.8)	0.9 (0.8, 1.2)*^#^	0.8 (0.6, 1.0)*^#^	1.5 (1.0, 2.3)^※^^§^	**<0.001**
Ciprofol requirements (mg·kg^-1^· min^-1^)	0.055 (0.487, 0.073)	0.058 (0.052, 0.068)	0.042 (0.031, 0.049)*^#^	0.045 (0.038, 0.050)*^#^	0.041 (0.032, 0.056)*^#^	**<0.001**
Ciprofol requirements (mg·kg^-1^)	1.046 (0.861, 1.427)	1.082 (0.853, 1.271)	0.795 (0.707, 0.889)*^#^	0.799 (0.601, 0.913)*^#^	0.789 (0.660, 0.968)*^#^	**<0.001**
Slope (95%CI)	0.031 (0.017, 0.046)	0.029 (0.016, 0.043)	0.021 (0.011, 0.030)*^#^	0.026 (0.019, 0.034)*^#^	0.019 (0.014, 0.023)*^#^	**<0.001**
Other outcomes						
Effective number of patients (%)	12 (42.8)	8 (28.5)*	15 (53.6)^#^	22 (78.5)*^#^^※^	25 (89.2)*^#^^※^^§^	**<0.001**
Use of NSAIDs	8 (28.6)	7 (25.0)	3 (10.7)	5 (17.9)	4 (14.3)	0.413
Use of opioids	0 (0)	0 (0)	0 (0)	0 (0)	0 (0)	>0.999
Use of other painkillers	0 (0)	0 (0)	0 (0)	0 (0)	0 (0)	>0.999
Overall satisfaction score (point)^a^	10 (10, 10)	10 (10, 10)	10 (10, 10)	10 (10, 10)	10 (10, 10)	0.254
Modified Aldrete score	10 (9, 10)	10 (9, 10)	10 (9, 10)	10 (9, 10)	9.5 (9.0, 10)	0.526
Length of PACU stay (min)	27(24, 35)	28 (25, 33)	32 (24, 39)	30 (24, 40)	29 (24, 36)	0.894
Postoperative complications^b^	0 (0)	0 (0)	0 (0)	0 (0)	0 (0)	>0.999
Length of hospital stay after surgery (day)	2.0 (1.0, 2.0)	2.0 (1.0, 2.0)	2.0 (1.0, 2.0)	2.0 (1.0, 2.0)	2.0 (1.0, 2.0)	0.813

Data are presented as mean (95% CI), median (inter-quartile range), or N (%). *P* values in bold indicate <0.05

*PACU* post-anesthesia care unit

^a^Scale range from 0 to 10 (0 represents the worst satisfaction and 10 represents the best satisfaction)

^b^Generally defined as new-onset medical conditions that were harmful to patients' recovery and required medical intervention, i.e., grade II or higher on the Clavien-Dindo classification^1^

*P <0.005, compared with Sufentanil 0.15μg×kg^-1^ after Bonferroni correction

^#^*P *<0.005, compared with Alfentanil 8 μg×kg^-1^ after Bonferroni correction

^※^*P* <0.005, compared with Alfentanil 10 μg×kg^-1^ after Bonferroni correction

^§^*P* <0.005, compared with Alfentanil 12 μg×kg^-1^ after Bonferroni correction

**Fig. 5:**
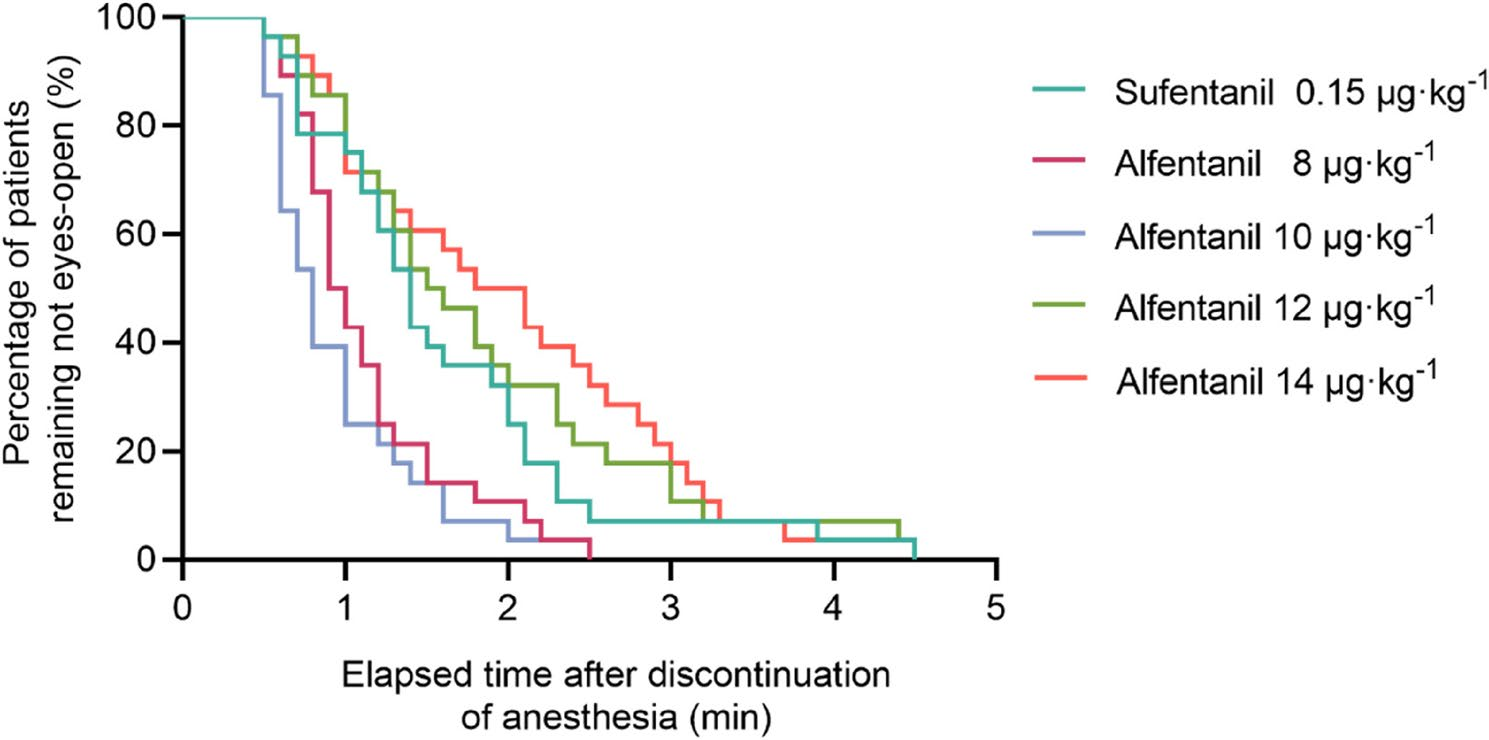
Cumulative percentages of patients remaining unconscious after discontinuation of ciprofol infusion in alfentanil group of 8 μg·kg^-1^, 10 μg·kg^-1^, 12 μg·kg^-1^, 14 μg·kg^-1^, and 0.15 mg×kg^-1^ sufentanil Group (area within different colors lines), using the Kaplan–Meier survival analysis

Emergence time was lower in Group A-10 (0.9 [0.8 to 1.2] min), 12 µg·kg^−1^ (0.8 [0.6 to 1.0] min) than those in Group A-8 (1.9 [1.0 to 2.8] min) and Group A-14 (1.5 [1.0 to 2.3] min), and Group S (1.4 [1.0 to 2.0] min) (*P* < 0.001;Table 2). The cumulative percentages of patients remaining unconscious after discontinuation of ciprofol infusion for five groups are displayed in Table 2; Fig. 5.

The VAS score of pain at the time of 30 min and 1 h after surgery was lower in Group A-10, Group A-12, and Group A-14 when compared with Group A-8 and Group S (*P* < 0.001), while the VAS score of pain at the time of 6 h after surgery, 12 h after surgery, and 1 d after surgery did not differ among the five groups (Table 3). The Ramsay sedation scale score at the time of 30 min and 1 h after surgery was higher in Group A-12 and Group A-14 when compared with Group A-8 and Group A-10 (*P* < 0.001), while the Ramsay sedation scale score was lower in Group A-8 and Group A-10 when compared with Group S (*P* < 0.001). The Ramsay sedation scale score at the time of 6 h after surgery did not differ among the five groups (Table 3).

**Table 3:** Visual analogue scale of pain (secondary outcomes) and Ramsay sedation scale during the perioperative period

	Sufentanil 0.15 µg⋅kg^−1^ (*n* = 28)	Alfentanil 8 µg⋅kg^−1^ (*n* = 28)	Alfentanil 10 µg⋅kg^−1^ (*n* = 28)	Alfentanil 12 µg⋅kg^−1^ (*n* = 28)	Alfentanil 14 µg⋅kg^−1^ (*n* = 28)	*P*-value
Visual analogue scale of pain, median (IQR), point^a^						
30 min after surgery	2 (2, 3)	3 (2, 3)	0 (0, 1)*^#^	0 (0, 1)*^#^	0 (0, 1)*^#^	**< 0.001**
1 h after surgery	1 (1, 2)	1 (0, 2)	0 (0, 1)*^#^	0 (0, 1)*^#^	0 (0, 1)*^#^	**< 0.001**
6 h after surgery	1 (0, 1)	1 (1, 1)	1 (0, 1)	1 (0, 2)	1 (1, 1)	0.369
12 h after surgery	0 (0, 1)	0 (0, 1)	1 (0, 1)	1 (0, 1)	0 (0, 1)	0.496
1 d after surgery	0 (0, 0)	0 (0, 0)	0 (0, 0)	0 (0, 0)	0 (0, 0)	> 0.999
Ramsay sedation scale, median (IQR), point^b^						
30 min after surgery	2 (2, 3)	2 (2, 2)*	2 (2, 2)*	3 (2, 3)^#※^	3 (2, 3)^#※^	**< 0.001**
1 h after surgery	3 (3, 3)	2 (2, 2)*	2 (2, 3)*	3 (3, 3)^#※^	3 (3, 3)^#※^	**< 0.001**
6 h after surgery	2 (2, 2)	2 (2, 2)	2 (2, 2)	2 (2, 2)	2 (2, 2)	0.256

Data are median (inter-quartile range). *P* values in bold indicate < 0.05

^a^ Score ranges from 0 to 10, where 0 = no pain and 10 = the worst pain

^b^ Ramsay sedation score ranges from 1 (restlessness) to 6 (deeply asleep and does not respond) and 2 indicates completely awake, quiet and cooperative

*P < 0.005, compared with Sufentanil 0.15 µg⋅kg^−1^ after Bonferroni correction

^#^*P* < 0.005, compared with Alfentanil 8 µg⋅kg^−1^ after Bonferroni correction

^※^*P* < 0.005, compared with Alfentanil 10 µg⋅kg^−1^ after Bonferroni correction

Effective number of patients was higher in Group A-10, Group A-12, and Group A-14 than those in Group A-8 (*P* < 0.001; Table 2). There was no difference in the use of NSAIDs, opioids, and other painkillers, overall satisfaction score, Modified Aldrete score, length of PACU stay, hospital stay after surgery and postoperative complications among five groups (*P* > 0.05; Table 2). Both MAP and HR at the time of loss of consciousness, immediately after cervical dilation, discontinuation of ciprofol, end of surgery, regain consciousness were higher in Group A-10 than those in Group A-8, Group A-12, and Group A-14, and Group S *P* < 0.001; Supplemental Table S1). SpO_2_ at the time of loss of consciousness was lower in Group A-14 (98 [94 to 100] %) than those in Group A-8 (100 [100 to 100] %), Group A-10.

(100 [98 to 100] %), Group A-12 (100 [99 to 100] %), and Group S (100 [100 to 100] %) (*P* < 0.001; Supplemental Table S1).

### Safety outcomes

The incidence of intraoperative hypotension was lower in Group A-8, Group A-10, Group A-12, and Group A-14 than those in Group S (*P =* 0.044; Table 4). The incidence of intraoperative desaturation was higher in Group A-14 than those in Group A-8, Group A-10, Group A-12, and Group S (*P* < 0.001; Table 4). The incidences of the other intraoperative and postoperative side effects including bradycardia, tachycardia, hypertension, nausea and vomiting, shivering, myotonia, abdominal bloating, constipation, and pruritus did not differ among five groups (*P* > 0.05; Table 4).

**Table 4:** Side effects

	Sufentanil 0.15 µg⋅kg^−1^ (*n* = 28)	Alfentanil 8 µg⋅kg^−1^ (*n* = 28)	Alfentanil 10 µg⋅kg^−1^ (*n* = 28)	Alfentanil 12 µg⋅kg^−1^ (*n* = 28)	Alfentanil 14 µg⋅kg^−1^ (*n* = 28)	*P*-value
Intraoperative adverse events						
Tachycardia^a^	3 (10.7)	5 (17.9)	0 (0.0)	1 (3.6)	2 (7.1)	0.121
Bradycardia^b^	2 (7.1)	1 (3.6)	3 (10.7)	3 (10.7)	4(14.3)	0.698
Hypertension^c^	3 (10.7)	2 (7.1)	1 (3.6)	1 (3.6)	1 (3.6)	0.713
Hypotension^d^	10 (35.7)	3(10.7)*	1 (3.6)*	2 (7.1)*	3 (10.7)*	**0.044**
Desaturation^e^	3 (10.7)	2 (7.1)	5 (17.9)	6 (21.4)	12 (42.9)*^#※§^	**< 0.001**
Nausea and vomiting	0 (0)	0 (0)	0 (0)	0 (0)	0 (0)	> 0.999
Shivering	2 (7.1)	1 (3.6)	0 (0)	1 (3.6)	0 (0)	0.882
myotonia	1 (3.6)	0 (0)	0 (0)	0 (0)	0 (0)	0.842
Postoperative adverse events						
Tachycardia^a^	0 (0)	0 (0)	0 (0)	0 (0)	0 (0)	> 0.999
Bradycardia^b^	0 (0)	0 (0)	0 (0)	0 (0)	0 (0)	> 0.999
Hypertension^c^	0 (0)	0 (0)	0 (0)	0 (0)	0 (0)	> 0.999
Hypotension^d^	0 (0)	0 (0)	0 (0)	0 (0)	0 (0)	> 0.999
Desaturation^e^	0 (0)	0 (0)	0 (0)	0 (0)	0 (0)	> 0.999
Nausea and vomiting	2 (7.1)	0 (0.0)	1 (3.6)	1 (3.6)	3 (10.7)	0.418
Shivering	3 (10.7)	2 (7.1)	0 (0)	1 (3.6)	2 (7.1)	0.486
Myotonia	0 (0)	0 (0)	0 (0)	0 (0)	0 (0)	> 0.999
Abdominal bloating	0 (0)	0 (0)	0 (0)	0 (0)	0 (0)	> 0.999
Constipation	0 (0)	0 (0)	0 (0)	0 (0)	0 (0)	> 0.999
Pruritus	0 (0)	0 (0)	0 (0)	0 (0)	0 (0)	> 0.999

Data are presented as N (%)

*PACU* post-anesthesia care unit

^a^Defined as heart rate > 100 beats per minute or an increase of > 20% from baseline

^b^Defined as heart rate < 60 beats per minute or a decrease of > 20% from baseline

^c^Defined as systolic blood pressure > 160 mmHg or an increase of > 30% from baseline

^d^Defined as systolic blood pressure < 90 mmHg or a decrease of > 30% from baseline

^e^Defined as pulse oxygen saturation less than 90% or a decrease of more than 5% (absolute value) from baseline

*P < 0.005, compared with Sufentanil0.15 µg⋅kg^−1^ after Bonferroni correction

^#^*P* < 0.005, compared with Alfentanil 8 µg⋅kg^−1^ after Bonferroni correction

^※^*P* < 0.005, compared with Alfentanil 10 µg⋅kg^−1^ after Bonferroni correction

^§^*P* < 0.005, compared with Alfentanil 12 µg⋅kg^−1^ after Bonferroni correction

## Discussion

In our present study, we determined the ED_50_ and ED_95_ of alfentanil attenuating the response to cervical dilation during hysteroscopy combined with ciprofol by the probit regression analysis and investigated the effect of alfentanil on reducing ciprofol requirement during hysteroscopy. Subsequently, our finding revealed that the calculated ED_50_ and ED_95_ of alfentanil were 9.73 [95% CI 8.57 to 10.56] µg·kg^−1^ and 15.02 [95% CI 13.57 to 18.12] µg·kg^−1^, respectively, and 10–12 µg·kg^−1^ alfentanil was associated with obvious ciprofol-sparing effect, with faster recovery from anesthesia, better postoperative analgesia, and decreased unhoped cardiovascular events.

Alfentanil is a new synthetic opioid analgesic that acts on specific µ opioid receptor, with the fastest onset of action, the most rapid time to peak effect, and the shortest distribution and elimination half-life, followed by sufentanil and fentanyl. Compared to fentanyl and sufentanil, alfentanil as a smaller distribution volume and systemic clearance, so the duration of action is the shortest [6, 21]. In a previous dose-response research using the modified Dixon’s up-and-down method, ED_50_ of alfentanil was 10.7 ± 2.1 µg·kg^−1^ to provide successful intubating conditions following inhalation induction using 5% sevoflurane and 60% nitrous oxide in oxygen, without neuromuscular blocking agents in day-case anesthesia [22]. Interestingly, another up-and-down sequential allocation trial further found that the ED_50_ of alfentanil required to inhibit the bronchoscopy reaction in females and males was 13.68 ± 4.75 and 17.96 ± 3.45 µg·kg^−1^ when combined with propofol administration during painless bronchoscopy with i-gel supraglottic airway device [23]. Our observation showed that the calculated ED_50_ and ED_95_ of alfentanil were 9.73 [95% CI 8.57 to 10.56] µg·kg^−1^ and 15.02 [95% CI 13.57 to 18.12] µg·kg^−1^ during hysteroscopy procedure, suggesting that when combined with ciprofol, a certain dose of alfentanil can express a strong analgesic and sedative effect, and effectively resist the response of patients with strong nociceptive stimulation caused by surgery or treatment.

Ciprofol is principally an agonist of the γ-aminobutyric acid type A receptor; it comprises the active ingredient HSK3486, which is similar to the currently used intravenous anesthetic propofol in clinical practice [24]. Increasing clinical trials have suggested that compared with propofol, ciprofol provided a better sedative efficacy, significantly shortened the time to fall asleep, improved the postoperative recovery quality and satisfaction, lowered the percentage of injection pain, nausea and vomiting, and the major cardiopulmonary and respiratory adverse events for painless endoscopy [25–27]. Furthermore, current evidences indicated that ciprofol combined with alfentanil provides a better sedative efficacy than propofol with higher recovery quality and satisfaction and significantly decreased respiratory depression events during painless endoscopic procedure [27, 28]. However, in this regard, rare established evidence focused on the effect of alfentanil on the ciprofol-sparing in patients underwent hysteroscopy procedure. In line with this, we conducted a preliminary explorationa and curiously found that alfentanil at doses from 10 to 14 µg·kg^−1^ had a significant ciprofol-sparing effect and the lower slope of the ciprofol requirements-duration curves when compared with 0.15 µg·kg^−1^ sufentanil and 8 µg·kg^−1^ alfentanil, indicating that when using 10–14 µg·kg^−1^ alfentanil, the influence of the operation duration on the demand for ciprofol was more moderate, that is, when the operation duration changed, the variation range of the demand for ciprofol was smaller. Based on all above evidences, alfentanil of 10–14 µg·kg^−1^ can be applied as a substitute to sufentanil anesthesia for operative hysteroscopic procedures. Due to the limited quality and quantity of the included studies, more high-quality studies are needed to validate these above conclusions and determine the optimal dose of alfentanil.

A previous work showed that alfentanil-propofol combination has more beneficial advantages over remifentanil and fentanyl in rapid onset and early recovery time (time to reach Modified Aldrete recovery score (MARS) to 8) and less respiratory depression for patients undergoing elective electrical cardioversion [29]. Accordingly, a recent report clarified that ciprofol combined with alfentanil can provide a better sedative efficacy, a higher post-endoscopic recovery quality and satisfaction than propofol in esophagogastroduodenoscopy followed by colonoscopy [27]. Additionally, Hu et al. investigated the anesthetic effect of different doses of alfentanil combined with ciprofol in elderly patients undergoing endoscopic retrograde cholangiopancreatography (ERCP) and found a statistically significant dose-related difference in recovery time [28]. Similarly, a recent meta-analysis pointed out that compared with propofol, alfentanil-propofol based sedative had presented shorter awakening time and directional force recovery time, with a lower prevalence rate of reported adverse event (i.e. nausea and vomiting, cough reflex, body movement, hypotension, and respiratory depression) for painless gastrointestinal endoscopy [8]. The present results of our study also proved that alfentanil at doses from 10 to 12 µg·kg^−1^ obviously shortened the emergence time from anesthesia when compared with 8 ang 14 µg·kg^−1^ alfentanil and 0.15 µg⋅kg^−1^ sufentanil, the possible reasons may be that the time to recovery from anesthesia was correlated with the dosage of alfentanil, while a small dose of alfentanil needs to be combined with a larger dose of ciprofol to achieve a certain depth of anesthesia to inhibit the response of patients and delayed the anesthesia recovery time. Conversely, the length of stay in PACU and hospital did not differ among five groups in our patients. Based on these above data, we provided the evidences that the dosage from 10 to 12 µg·kg^−1^ of alfentanil in combination with ciprofol may be the more suitable prescription composition for the postoperative awaking and recovery of patients during hysteroscopy.

It has been reported that patients with PCA treated in combination with alfentanil and morphine possessed a longer analgesic duration within 24 h, better analgesic effects at rest and during movement, without affecting the onset rate, and required less ketamine infusion for rescue than with fentanil during major surgery [30]. Another clinical trial stated that total intravenous anesthesia (TIVA) with and propofol and alfentanil remarkably reduced VAS scores of pain at 30 min and 60 min and provided early postoperative pain control than propofol and remifentanil in patients scheduled for lumbar discectomy [31]. In additional, a prospective, randomized, double-blind study illustrated that intravenous PCA with alfentanil and oxycodone were similar in VAS scores of pain, the cumulative PCA dose, adverse effects, sedation level and satisfaction within postoperative 48 h in patients undergoing laparoscopic cholecystectomy [32]. The results of this study show that the VAS scores of pain of patients administered alfentanil at 10–14 µg·kg^−1^ was lower than that of 8 µg·kg^−1^ alfentanil and 0.15 µg⋅kg^−1^ sufentanil, and there were no differences at other time points. This suggests that the administration regimen not only has better intraoperative analgesic and sedative effects, but also has better postoperative analgesic quality. However, the specific therapeutic effect remains to be further verified.

In a previous prospective, randomized, double-blind study, alfentanil effectively attenuated the pressor response to nasotracheal intubation and decreased the incidence of hypotension compared with remifentanil in patients undergoing elective maxillofacial surgery [10]. Ozkan et al. compared the effect of fentanyl, remifentanil and alfentanil in association with propofol and midazolam for elective electrical cardioversion and saw that the similar incidence of hypotension and bradycardia but less respiratory depression in propofol alfentanil combination [29]. A present meta-analysis showed that compared with propofol, alfentanil combined with propofol had a more stable hemodynamic and respiratory profile and a lower prevalence rate of reported adverse event (i.e. nausea and vomiting, cough reflex, body movement, hypotension, and respiratory depression) for painless gastrointestinal endoscopy [8]. Our current results that ciprofol combined alfentanil group at the dose of 8μg·kg^-1^, 10μg·kg^-1^, 12μg·kg-^1^ and 14μg·kg^-1^ did not increase the incidences of adverse events including nausea and vomiting, desaturation, hypotension, bradycardia, and arrhythmia when compared with the ciprofol combined sufentanil, which may be because of the using of the much lower dose of alfentanil and ciprofol in our study.Whether alfentanil-ciprofol combination has a superior safety profile in comparison with alfentanil-propofol requires further demonstration.

The present research still has some limitations. First of all, our study subjects were ASA I-II female patients, aged 20–40 years, and the optimal dosage of alfentanil and its effect on the consumption of ciprofol in male patients as well as gender differences need to be further verified. Secondly, we only determined the efficacy of alfentanil combined with ciprofol in the patient population undergoing hysteroscopic surgery. The efficacy and safety in other painless endoscopic examinations (such as gastroscopy, fiberoptic bronchoscopy, and colonoscopy) will be discussed in our subsequent designed studies. Third, we only clarified the benefits of different doses of alfentanil combined with ciprofol in reducing perioperative adverse events and improving immediate recovery after endoscopic surgery, but did not compare the effects of alfentanil combined with ciprofol on the early and long-term prognosis of patients, such as postoperative anxiety, delirium, sleep quality and neurocognitive function. Therefore, it is necessary to conduct more large-scale studies to elucidate. Fourth, compared with propofol, ciprofol has better anesthetic performance and fewer side effects such as injection pain, hypoxemia. However, based on the results of this study, the advantages of the combination of alfentanil and ciprofol in endoscopic surgery over the combination of alfentanil and propofol remain unclear and need further verification.

## Conclusions

During hysteroscopic surgery, compared with sufentanil, the total dosage of propofol given to women at 10–12 µg·kg^−1^was significantly reduced, the anesthesia recovery time was advanced, postoperative analgesia was improved, and hemodynamic adverse events were reduced. Whether alfentanil combined with ciprofol is superior to sufentanil in terms of prognosis for these population still requires further extensive and high-quality verification.

## Supplementary Information

Supplementary Material 1.

Supplementary Material 2.

## Data Availability

All information required is given in the text and supplementary materials, other supplementary information can be obtained upon email from the corresponding author.

## Abbreviations

ED_50_: Median effective dose
ED_95_: 95% effective dose
MAC: Monitored anesthesia care
CABG: Coronary artery bypass graft
ASA: American Society of Anesthesiologists
BMI: Body mass index
ECG: Electrocardiogram
SpO2: Pulse oximeter
NIBP: Noninvasive blood pressure
PETCO2: end-expiratory partial pressure of carbon dioxide
SE: state entropy
LOC: lost consciousness
PACU: post-anesthesia care unit
VAS: visual analogue scale
SBP: Systolic blood pressure
DBP: Diastolic blood pressure
HR: heart rate
VAS: visual analogue scale
MARS: Modified Aldrete recovery score
ERCP: endoscopic retrograde cholangiopancreatography
PCA: patient-controlled analgesia
TIVA: total intravenous anesthesia
