# Supplementary material for: The median Effective Dose (ED50) and the 95% Effective Dose (ED95) of alfentanil in inhibiting responses to cervical dilation when combined with ciprofol during hysteroscopic procedure: a prospective, double-blind, dose-finding clinical study
Source: BMC Anesthesiol. 2025 Jul 19;25:353. doi: 10.1186/s12871-025-03217-5 (PMC12275378; doi:10.1186/s12871-025-03217-5)
Supplement: Supplementary file 2 — Supplementary Material 2. [file 12871_2025_3217_MOESM2_ESM.docx]

**Cover Letter**

June 18, 2025

Guangde Tu, PhD Senior Editor

*BMC Anesthesiology*

Kirti Kamble, MD

Senior Editor

*BMC Anesthesiology*

**Manuscript:** Afentanil Decreased Ciprofol Requirement During Hysteroscopy: A Double-blind, Dose-response Study

**Manuscript Number:** baa1617e-2fc0-479c-90fd-22774465548c

Dear Dr. Tu and Dr. Kamble:

Thank you for giving us the opportunity to revise our manuscript in the title and number listed above.

After careful reading all comments from you and the reviewers, we now provided a revised version with changes high-lighted in red together with a point-by-point response. We added the Declarations section follow the Conclusions section, included a ‘Consent for publication’ section in the Declarations, added a statement to indicate that our study adheres to CONSORT guidelines, and included a statement on ethics approval.

We hope that the revision will be satisfactory to you the reviewers. Please tell us if we can do anything further.

Yours sincerely,

Li-Li Xu, MD, on behalf of all authors.

Department of Anesthesiology

Women’s Hospital, Zhejiang University School of Medicine,

Hangzhou, Zhejiang Province, 310006, China.

Phone: 86 571 87061007

Fax: 86 571 87061878

Email: xulili@zju.edu.cn
